# The Tip Position of Peripherally Inserted Central Catheters by the Sherlock 3CG System Was Almost Deeper Than Zone B: A Case Series

**DOI:** 10.7759/cureus.40711

**Published:** 2023-06-20

**Authors:** Mitsutaka Edanaga, Honami Sato, Gen Ochiai, Michiaki Yamakage

**Affiliations:** 1 Anesthesiology, Sapporo Medical University School of Medicine, Sapporo, JPN; 2 Anesthesiology, Kushiro City General Hospital, Kushiro, JPN; 3 Anesthesiology, Obihiro Kosei Hospital, Obihiro, JPN

**Keywords:** power picc, sherlock 3cg system, zone b, transesophageal echocardiography, peripherally-inserted central catheterization

## Abstract

A first analysis of deaths due to central venous catheterization (CVC) in Japan in 2017 reported peripherally inserted central catheterization (PICC) as an alternative to CVC. In 2018, Sherlock™ 3CG (C.R. Bard Inc., New Jersey, USA) and Power PICC^®^ became available for use in Japan. The electromagnetic mechanism of the Sherlock 3CG system often eliminates the need for the use of fluoroscopic devices, such as C-arm scanners. In this clinical report, we describe five cases of patients who underwent PICC guided by the Sherlock 3CG system and were evaluated by transesophageal echocardiography (TEE). The patients were adapted for PICC for highly invasive urologic, thoracic, and dental surgery. Also, the positions of the catheter tip were confirmed by TEE in all cases. The mean distance from the access vein to the catheter tip was 41.1 ± 3.8 cm. Chest X-ray analysis showed a mean distance of 40.0 ± 21.5 mm between the carina and catheter tip. Bicaval TEE views showed that the Power PICC tip had not been advanced into the right atrium in any of the cases.

We concluded that the tip positions of the Power PICC guided by the Sherlock 3CG system were almost deeper than Zone B and not in the right atrium.

## Introduction

A first analysis of deaths due to central venous catheterization (CVC) in Japan in 2017 [1] reported peripherally inserted central catheterization (PICC) as an alternative to CVC. The merits of PICC are the low incidence of catheter-related infection and the avoidance of serious complications, such as pneumothorax and hemothorax. The conventional PICC procedure requires a fluoroscopic device, such as a C-arm scanner. In 2018, a new navigation device, the Sherlock™ 3CG (C.R. Bard Ltd., New Jersey, USA), and Power PICC® became available for use in Japan. The Sherlock 3CG system uses an electromagnetic system to guide catheter tip positioning, eliminating the need for the use of a fluoroscopic device in most cases. In this report, we describe our experience with five cases in which the PICC was successfully inserted under Sherlock 3CG system guidance, as confirmed by transesophageal echocardiography (TEE) evaluation. The previous report [2] indicated that the catheter tip of the CVC has to be from the junction site of the bilateral innominate vein to the upper superior vena cava (Zone B). Zone B is recommended as the appropriate region of the CVC catheter tip in the 2017 Practical Guide for central venous catheterization of the Japanese Society of Anesthesiologists [3]. In this clinical report, we showed that the tip positions of the Power PICC inserted under Sherlock 3CG guidance were almost deeper than Zone B and not into the right atrium. Also, we would like to suggest that the tip positions of the Power PICC are in the safety region.

## Case presentation

After approval of the institutional review boards of Sapporo Medical University School of Medicine (302-22) and UMIN Clinical Trial Registration (UMIN000033775), informed consent was obtained from all five patients for study participation. The patients were scheduled to undergo PICC under general anesthesia before surgery for perioperative management of highly invasive urologic, thoracic, and oral surgery. Additionally, we planned to use TEE for the confirmation of the tip of the Power PICC. Table 1 shows the characteristics of the five patients. 

**Table 1:** Patients characteristics

Case	Case 1	Case 2	Case 3	Case 4	Case 5
Age (years)	76	73	62	67	72
Gender	Male	Male	Female	Female	Male
BMI (kg/m^2^)	24	23	27	22	24
Approach	Left	Right	Left	Left	Left

Sherlock 3™ CG system

Typically, when we perform CVC under general anesthesia, the right internal jugular vein is the first choice as the access site. However, during cranial, facial, oral, and neck surgery, a catheter inserted via the right internal jugular vein might interfere with the surgical procedure and be a source of catheter-induced infection. Additionally, according to the first analysis in 2017 mentioned above, it is thought that the importance of PICC as an alternative to CVC increases. In a clinical situation, we planned to use the Sherlock 3CG system and Power PICC [4]. The Sherlock 3CG system consists of a monitor and a Y sensor [5], and the Power PICC has a magnetic tip at the distal end of the catheter [6]. The Y sensor is placed on the chest wall [7] and helps track the magnetic tip of the Power PICC catheter. Additionally, intravascular ECG assessments [8] are used to accurately place the catheter tip at the cavo-atrial junction [9]. With the intravascular ECG system, the p-wave becomes more peaked when the catheter tip is at the cavo-atrial junction, but it reveals a biphasic pattern when the tip is at the right atrium. We planned to fix the catheter tip at the position at which the p-wave became peaked, and a biphasic pattern was not visualized. According to the concept of the Sherlock 3CG system, patients on whom it is planned to perform the Sherlock 3CG-guided method of PICC may not need C-arm scanning or chest X-rays. However, all our patients were scheduled to undergo chest X-rays for confirmation of appropriate tip placement and to obtain images. Additionally, we planned to use TEE to evaluate whether the catheter tip had been advanced into the right atrium and to assess the reliability of the Sherlock 3CG system. Before PICC, in all patients, the arm on the side of the puncture was extended out to 90 degrees relative to the chest. We selected the basilic vein as the primary access site. The guidewire and sheath were inserted after the puncture of the vein, and the first access region was a basilic vein. After the puncture of the vein, the guidewire and sheath were inserted. If the guidewire was not successfully advanced into the basilic vein, we next tried the same procedure via the brachial vein as the secondary access site. Next, the Power PICC was inserted through the sheath, and the catheter was advanced to the appropriate position by using the Y sensor and monitor of the Sherlock 3CG system.

Results

**Figure 1:**
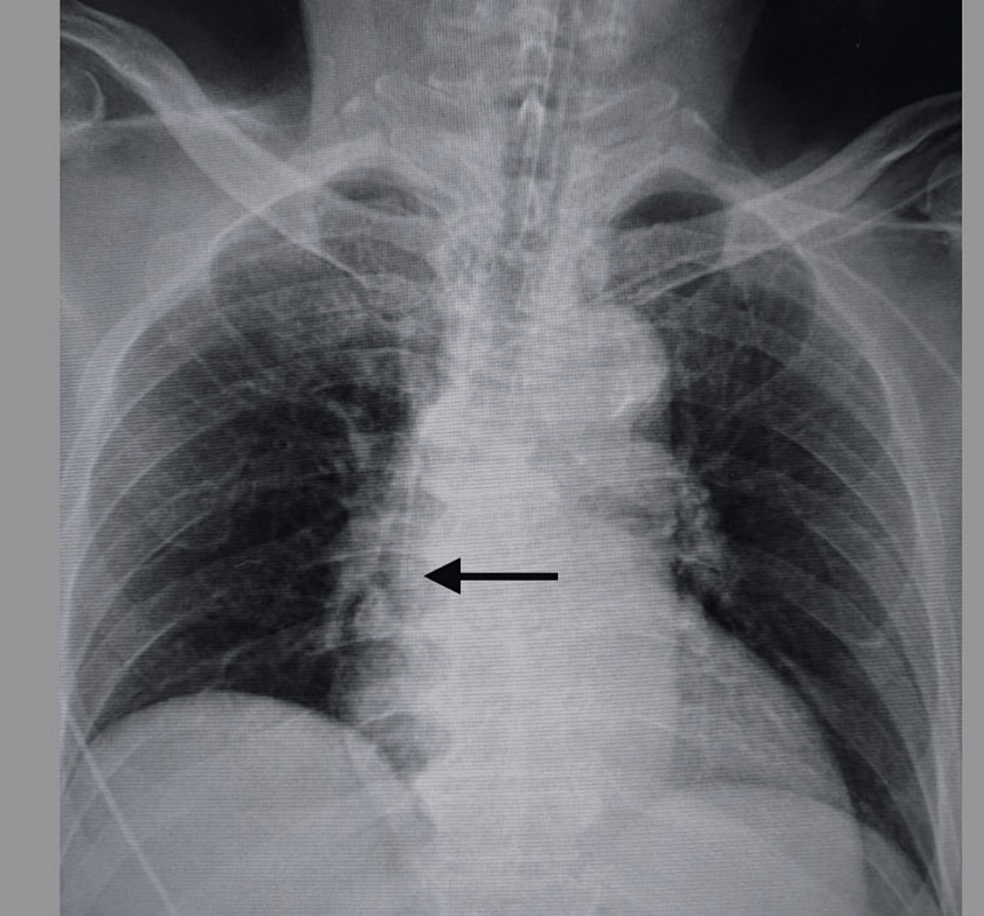
Chest X-ray after insertion of the Power PICC The black arrow was the catheter tip of the Power PICC guided by the Sherlock 3CG system in Case 1.

**Figure 2:**
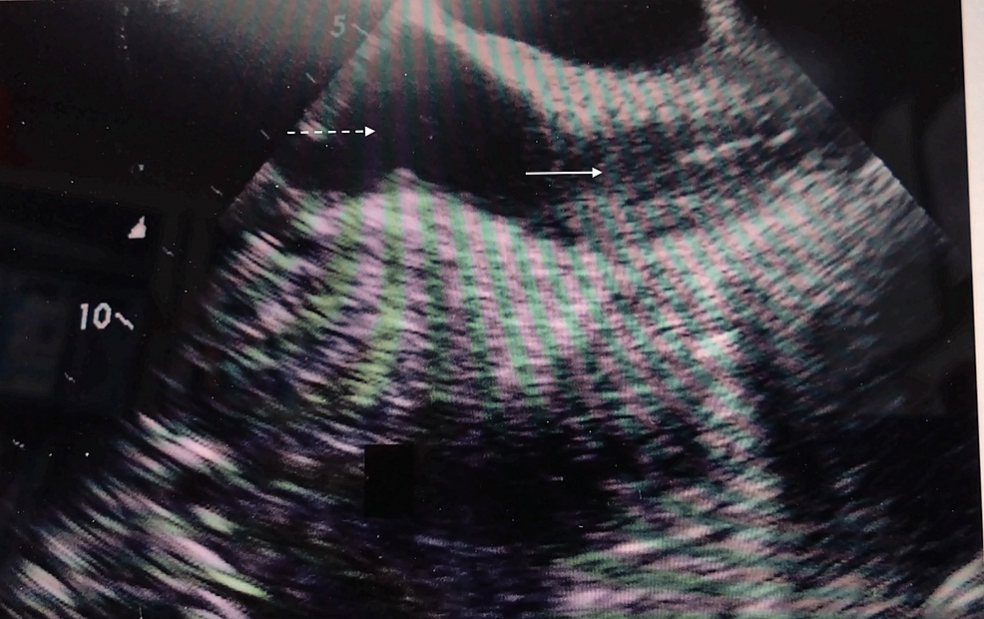
Bicaval transesophageal echocardiography views after insertion of the Power PICC in the five patients. The white arrow shows the superior vena cava, and the white dotted line shows the right arial.

**Figure 3:**
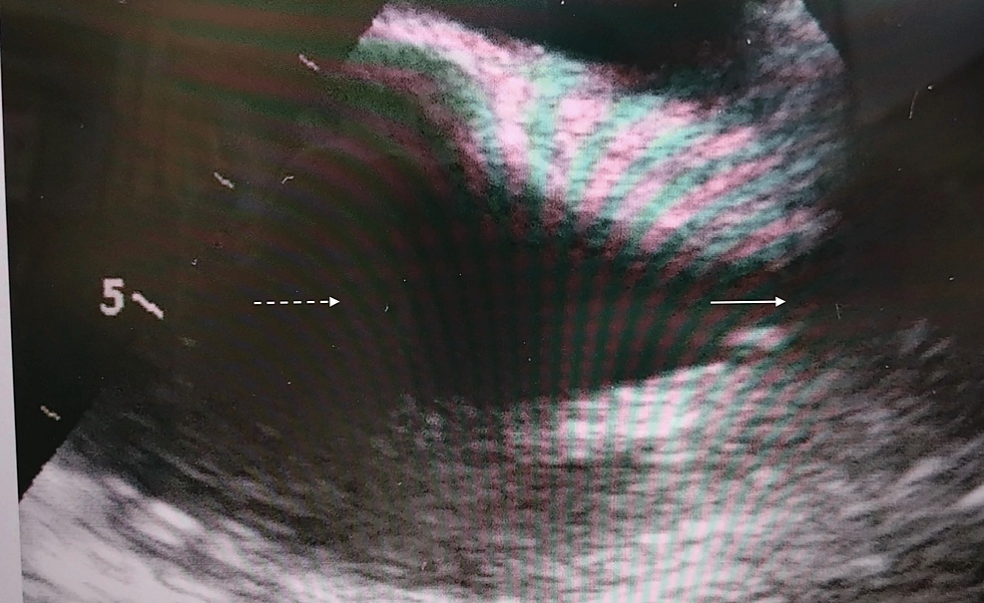
Bicaval transesophageal echocardiography views after insertion of the Power PICC in Case 2. The white arrow shows the superior vena cava, and the white dotted line shows the right arial.

**Figure 4:**
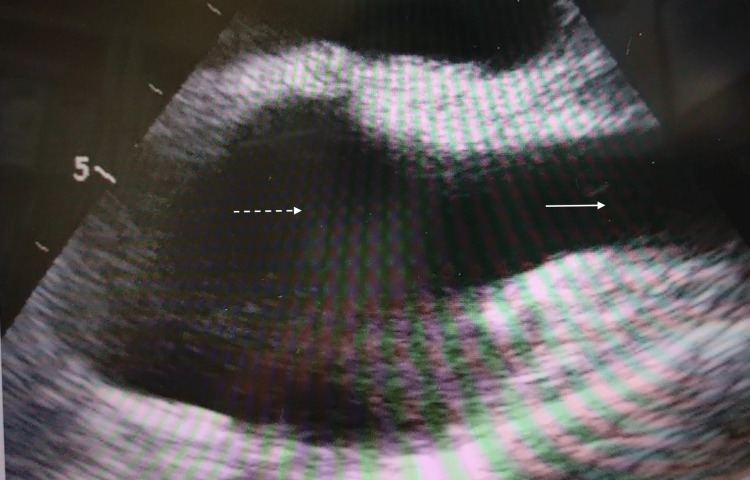
Bicaval transesophageal echocardiography views after insertion of the Power PICC in Case 3. The white arrow shows the superior vena cava, and the white dotted line shows the right arial.

**Figure 5:**
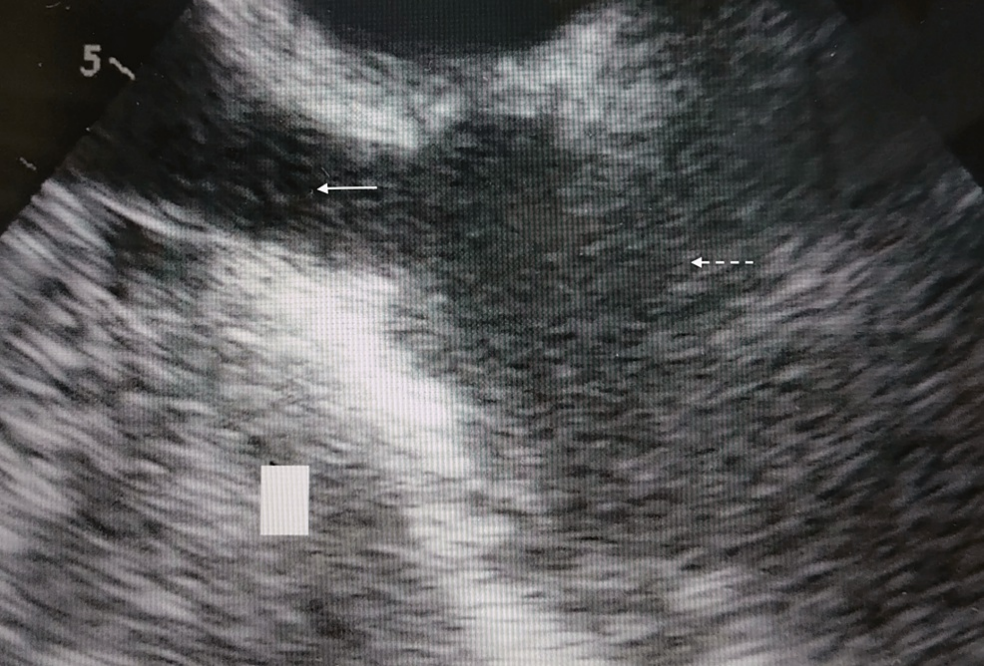
Bicaval transesophageal echocardiography views after insertion of the Power PICC in Case 4. The white arrow shows the superior vena cava, and the white dotted line shows the right arial.

**Figure 6:**
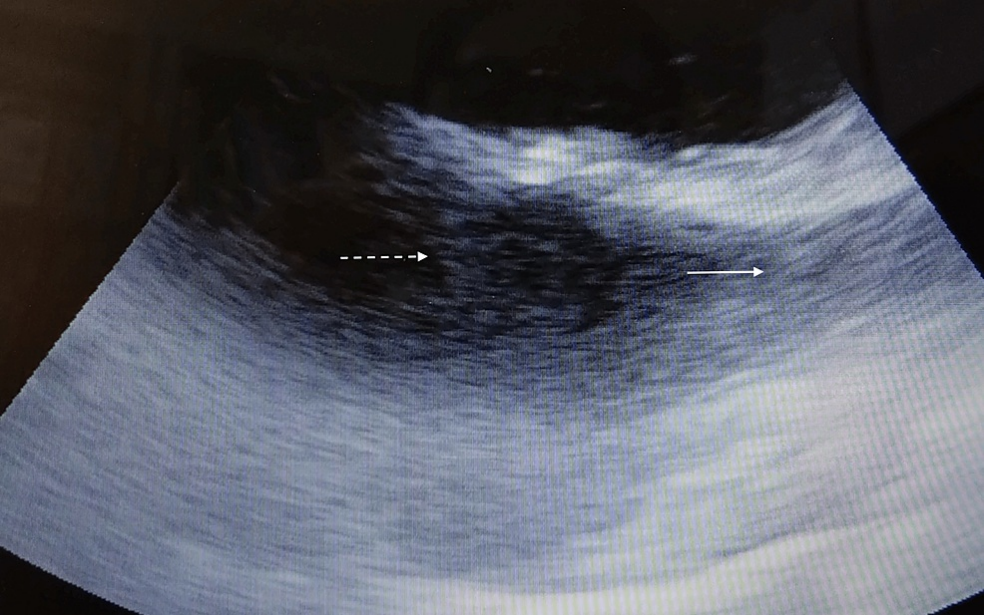
Bicaval transesophageal echocardiography views after insertion of the Power PICC in Case 5. The white arrow shows the superior vena cava, and the white dotted line shows the right arial.

The reasons for performing PICC in the five patients were highly invasive urologic (n=2), thoracic (n=1), and dental (n=2) surgeries. The catheters were inserted via the left arm in four patients and the right arm in one patient. Patients’ ages ranged from 62 to 76 years, three were male, and two were female. All procedures were successfully completed, and the catheters were advanced to the appropriate position around the cavo-atrial junction (Figure 1). The results of the procedures are shown in Table 2. The mean distance from the access vein to the catheter tip was 41.1 ± 3.8 cm. In the post-insertion chest X-ray analyses, the tips of the catheter almost exceeded the carina in all cases. The mean distances from the carina to the catheter tip were 40.0 ± 21.5 mm. Subsequently, TEE was performed on all patients. Bicaval TEE views showed that the tips of the Power PICC were not advanced into the right atrium in any of the patients (Figures 2-6). Additionally, there were no complications after catheterization, such as supraventricular arrhythmia, pneumothorax, hemothorax, cardiac tamponade, and catheter-induced thrombosis, in any of the patients.

None of the catheters (Figure 2: Case 1, Figure 3: Case 2, Figure 4: Case 3, Figure 5: Case 4, Figure 6: Case 5) had entered the right atrium.

**Table 2:** Results of peripherally inserted central catheterization

Case	Case 1	Case 2	Case 3	Case 4	Case 5
Distance from the access vein to the catheter tip (cm)	44.5	41	41	35	44
Distance from the carina to the catheter tip (mm)	47.1	26.8	56.3	9.4	60.6
Whether the catheter tip had entered the right atrium (Yes or No)	No	No	No	No	No

## Discussion

In this clinical report, we would mainly like to emphasize the two results. First, we found that the catheter tips of the Power PICC-inserted Sherlock 3CG system guidance were positioned almost deeper than Zone B. Our results indicated that Sherlock 3CG system-guided PICC would be safe and useful. According to the 2017 practical guide for central venous catheterization of the Japanese Society of Anesthesiologists [3], Zone B is recommended as the appropriate position of the CVC. In our clinical report, the tips of the Power PICC inserted under Sherlock 3CG guidance were positioned around the cavo-atrial junction. Thus, the positions of the Power PICC were in the lower third of the superior vena cava, around the cavo-atrial junction, as has also been reported [10]. It was reported that displacement of the central venous catheter from the cavo-atrial junction was associated with a high incidence of catheter malfunction [11]. Additionally, maintaining the PICC tip in the lower one-third of the superior vena cava significantly reduces the incidence of catheter-related infections [12]. In the intravascular ECG system, the p-wave became more peaked when the catheter tip was positioned not at the right atrium but at the cavo-atrial junction. As a result, chest X-rays in all patients in this study showed that the tips of the Power PICC were almost deeper than the level of the carina (Zone B). 

Second, the TEE evaluation confirmed that the catheter tips were not advanced into the right atrium. The bicaval view of TEE was used to visualize the right atrium. Hence, our case reports indicated that TEE was useful for the confirmation of the position of the catheter tip.

## Conclusions

Considering the results of our case series and previous reports, we concluded that Power PICC insertion guided by the Sherlock 3CG system is a safety and accurate procedure and that the positioning of the tip of the Power PICC was deeper than Zone B but not in the right atrium. The appropriate position of the catheter tip in the general CVC may need to be reconsidered in the future.
